# Case Report: Gastric Mucormycosis- a rare but important differential diagnosis of upper gastrointestinal bleeding in an area of Helicobacter pylori endemicity

**DOI:** 10.12688/wellcomeopenres.15026.2

**Published:** 2019-03-27

**Authors:** Sudeep Adhikari, Ajaya Raj Gautam, Buddhi Paudyal, Keshav Raj Sigdel, Buddha Basnyat

**Affiliations:** 1 Internal Medicine, Patan Academy of Health Sciences, Kathmandu, Nepal; 2 Oxford University Clinical Research Unit, Patan Hospital, Kathmandu, Nepal

**Keywords:** gastric mucormycosis, mucormycosis, helicobacter pylori, peptic ulcer disease, upper gastrointestinal disease

## Abstract

A 57 years female from the hills of Nepal presented with upper gastrointestinal bleeding with gastric ulcer evident on endoscopy. Though initially treated with *Helicobacter pylori* ( *H. pylori*) eradication therapy alone, biopsy later on revealed both mucormycosis and *H. pylori* infection. She was then treated with antifungals liposomal amphotericin B followed by posaconazole which led to complete recovery. Mucormycosis is a rare but life-threatening fungal disease of immunocompromised host though our patient was immunocompetent. If recognized and treated at early stage, as in our patient, prognosis is good. A high index of suspicion is required for considering this disease in *H. pylori* endemic regions such as Nepal, and is crucial for early recognition and treatment.

## Introduction

Upper gastrointestinal bleeding is a common medical emergency which is usually treated with initial stabilization followed by upper gastrointestinal endoscopy. Peptic ulcer disease is a common finding in endoscopy which is usually associated with infection with *Helicobacter pylori* ( *H. pylori*) ^1^. In a developing country such as Nepal, *H. pylori* infection is so common ^2^ that treatment with two antibiotics and one proton pump inhibitor therapy, popularly called as ‘triple therapy’, is usually started empirically for eradication even before the presence of the infection is established. But there can be instances when some rare disease can be the cause of ulcers and bleeding, which if overlooked may lead to a fatal outcome without proper treatment. Here we present a case of upper gastrointestinal bleeding diagnosed as gastric mucormycosis.

Mucormycosis is a rare and often a life-threatening fungal disease, caused by a mold of the order Mucorales, characterized by vascular invasion by hyphae with thrombosis and necrosis ^3^. This is classically a fatal disease of immunocompromised state, with rhino-cerebral, pulmonary and cutaneous being the common types ^4^. Contrary to the classical description of this disease, our patient with gastric mucormycosis was an immunocompetent individual who recovered after antifungal treatment.

## Case report

A 57 year old female from Sindhuli, Nepal, with no known medical comorbidities other than smoking, presented to the emergency department with two episodes of hematemesis over 24 hours. She had a 4-month history of dyspepsia, melena and fatigability. On physical exam, she was pale, blood pressure was 90/50 mm Hg with a heart rate of 130 beats per minute. Other examinations, including abdominal exam, were unremarkable. She was resuscitated with intravenous fluids and packed cell transfusion.

Laboratory parameters with normal ranges in parenthesis, are as follow:

Complete blood count before transfusion: white cell count 7.9 (4–10) × 10 ^9^/L; neutrophils 70%; lymphocytes 26%; monocytes 4%; red blood cells 2.8 (4.2–5.4) × 10 ^12^/L; haemoglobin 9 (12–15) g/dL; platelets 295 (150–400) × 10 ^9^/L.

Biochemistry: random blood sugar 124 (65–110) mg/dL; urea 49 (17–45) mg/dL; creatinine 0.9 (0.8–1.3) mg/dL; sodium 140 (135–145) mmol/L and potassium 4 (3.5–5) mmol/L.

Hepatic panel: bilirubin total 1 (0.1–1.2) mg/dL and direct 0.6 (0–0.4) mg/dL; alanine transaminase 35 (5–30) units/L; aspartate transaminase 40 (5–30) units/L; alkaline phosphatase 98 (50–100) IU/L; albumin 3.5 (3.5–5) g/dL

After stabilization, she underwent upper gastrointestinal endoscopy the same day which revealed an ulcer (10 mm × 6 mm) in lesser curvature of the stomach without active bleeding. The provisional diagnosis was gastric ulcer due to *H. pylori* infection. The main differential diagnosis was gastric carcinoma, hence biopsy was taken from the ulcer.

**Figure 1.**
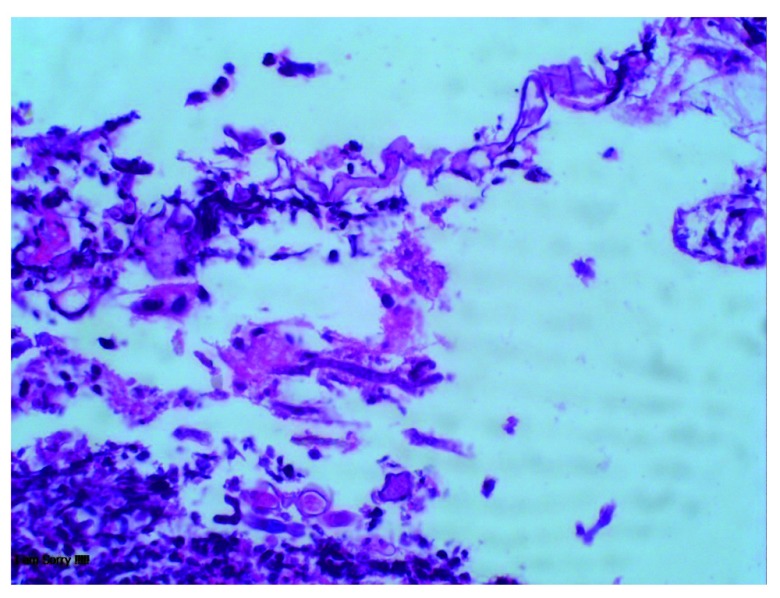
Histopathology examination of ulcer showing fungi with broad ribbon like morphology and fruiting bodies consistent with mucor.

She was started on triple therapy regimen empirically for *H. pylori* eradication containing clarithromycin 500 mg, amoxicillin 1 gm and pantoprazole 40 mg twice daily for 14 days, and discharged. When she returned for follow-up, the histopathology showed fungi with broad ribbon like morphology, fruiting bodies consistent with mucor in the ulcer, as well as in granulation tissue but no necrosis or vascular thrombi ( Figure 1). It was also positive for *H. pylori* in Giemsa staining. She was diagnosed with gastric mucormycosis with *H. pylori* coinfection. But the confirmation of mucormycosis via culture or molecular tests could not be done because of unavailability of these tests in our facility. The abdominal imaging was not done because there was no evidence of invasive form of the disease in histopathology.

She was readmitted and started on liposomal amphotericin B, 5 mg/kg on the first day followed by 10 mg/kg for the next 13 days. She completed a total of 2 weeks of therapy without any complication. She had no dyspepsia, melena or hematemesis by then. She was then discharged with posaconazole 300 mg once daily, as an oral step-down therapy. However the blood level of posaconazole to ensure the therapeutic level was not tested due to its unavailability in resource- limited setup in Nepal.

On repeat follow up in 1 month, she was asymptomatic. Repeat endoscopy showed a healing ulcer in lesser curvature of stomach. Repeat biopsy showed absence of fungal hyphae. She took posaconazole for total 2 months and stopped the medicine due to financial constraints. She was doing well when she was last followed up 4 months later.

## Discussion

Our patient presented with upper gastrointestinal bleed, and received H. pylori eradication treatment empirically after ulcer was evident on endoscopy. This is standard practice in Nepal as prevalence of *H. pylori* infection is very high in the general population ^2^, and it is also not always possible to perform tests for *H. pylori* detection as the tests are either unavailable or unaffordable. Fortunately, we took a gastric biopsy to try to rule out gastric malignancy. The biopsy serendipitously helped to clinch the proper diagnosis of gastric mucormycosis, which otherwise would not have been possible. Though our patient had coinfection with *H. pylori*, the diagnosis of mucormycosis became alarming because of its high fatality, and the need of specific antifungal treatment with amphotericin B.

Mucormycosis is a life threatening disease occurring in immunocompromised patients. The known risk factors for this disease include diabetes mellitus, particularly with ketoacidosis, treatment with glucocorticoids, hematologic malignancies, hematopoietic cell or solid organ transplantation, treatment with deferoxamine, iron overload, AIDS, injection drug use, trauma/burns, premature birth and malnutrition ^5^. But our patient had none of these risk factors, yet suffered from the disease.

The common sites of mucormycosis are the sinuses (39%), lungs (24%) and skin (19%). The gastrointestinal tract is involved in only 7% of patients, with the stomach being the most common site. Gastrointestinal mucormycosis is usually fatal (85% mortality) ^4, 6^. It can be classified into three forms- colonization of pre-existing ulcer, infiltration, and vascular invasion, with increasing fatality in this order ^7^. Our patient had early infiltrative form of the disease, without vascular invasion. Surgical debridement and antifungal therapy remain the mainstay of therapy, but we treated our patient with medical therapy alone. There are reports where early institution of antifungal therapy without the surgical intervention has led to the recovery in cases with non-invasive form of the disease like in our patient. Tathe *et al*. reported a case of gastric mucormycosis associated with gastric ulcer who recovered with antifungal treatment, and hypothesized that survival was due to detection and treatment at early nonfatal stage of the disease (colonization with early infiltration of pre-existing ulcer by the fungi rather than invasive ulcerative form of the disease which is almost always fatal) ^8^. Alfano *et al.* reported another case of gastric mucormycosis in a liver and kidney transplant recipient who recovered with antifungal therapy for total of 6 months ^9^. However our patient could not continue the medicine for more than 2 months due to financial constraints.

While transitioning the amphotericin to posaconazole, they should be overlapped until the therapeutic level of posaconazole is achieved in blood ^10^. But testing of blood level of the drug is not available in Nepal, hence the overlapping was not done. The resource limitation led to many pitfalls during management of our patient, yet she recovered from the infection as evidenced by clinical and histopathological remission. The survival of our patient may be attributed to it being the non-invasive form of disease, the absence of necrosis and thrombosis, and possibly also because of early diagnosis and prompt treatment. However this therapeutic strategy in early form of disease needs to be established as a standard of care by further studies.

Though validation by epidemiological studies is yet to be done, the estimated burden of mucormycosis in Nepal has been reported as 0.2 per 100000 per year ^11^, while in India it is estimated at 0.14 per 100000 ^12^. A study in Nepal reported 3 cases of mucormycosis among 331 cases of benign sinonasal masses ^13^, but no account of the gastrointestinal form of disease has been documented in Nepal. The prevalence of mucormycosis appears to be low in Nepal, and we do not routinely consider its possibility in the differential diagnosis of peptic ulcer disease. Our case demonstrates the obvious usefulness of doing a proper gastric histopathological study, where available, even in areas of *H. pylori* endemicity.

## Conclusion

Rare diseases like gastric mucormycosis may present as upper gastrointestinal bleeding, mimicking peptic ulcer disease. It may also co-exist with *H. pylori* infection. Mucormycosis is a life-threatening fungal disease of immunocompromised state, but it may rarely affect immunocompetent individuals too. Though usually fatal, recognition and treatment at its early stage may lead to recovery.

## Consent

Written informed consent for publication of clinical details and clinical images was obtained from the patient herself.

## Data availability

All data underlying the results are available as part of the article and no additional source data are required
