# Supplementary material for: Case Report: Gastric Mucormycosis- a rare but important differential diagnosis of upper gastrointestinal bleeding in an area of Helicobacter pylori endemicity
Source: Wellcome Open Res. 2019 Mar 27;4:5. Originally published 2019 Jan 17. [Version 2] doi: 10.12688/wellcomeopenres.15026.2 (PMC6518441; doi:10.12688/wellcomeopenres.15026.2)
Supplement: Peer review history

# Peer review: Reviewer response for version 2

**DOI:** 10.21956/wellcomeopenres.16593.r35145

**Published:** 2019-05-15

**Authors:** M. Veronica Dioverti

**Affiliations:** 1 John Hopkins University, Baltimore, MD, USA

Authors did a good job amending comments.

I have read this submission. I believe that I have an appropriate level of expertise to confirm that it is of an acceptable scientific standard.

# Peer review: Reviewer response for version 2

**DOI:** 10.21956/wellcomeopenres.16593.r35470

**Published:** 2019-05-13

**Authors:** Raju Pangeni

**Affiliations:** 1 HAMS Hospital, Kathmandu, Nepal

The authors have nicely presented an interesting case regarding an uncommon disease which is difficult to diagnose in a resource limited setting. Despite appropriate therapy, invasive mucormycosis itself is a deadly disease regardless of organ involved and requires surgical debridement in most cases.

 In the present case, the tissue/vascular invasion couldn't be demonstrated and the patient was treated against H Pylori and Mucor simultaneously. Hence, it may not be able to label mucor as the etiological agent behind bleeding gastric ulcer. In my opinion, Mucor should be regarded as a co-infection.

 However, this report makes a point that the endoscopists should consider biopsy among all patients with gastric ulcers.

I have read this submission. I believe that I have an appropriate level of expertise to confirm that it is of an acceptable scientific standard.

# Peer review: Reviewer response for version 2

**DOI:** 10.21956/wellcomeopenres.16593.r35144

**Published:** 2019-04-05

**Authors:** Gaetano Alfano

**Affiliations:** 1 Surgical, Medical and Dental Department of Morphological Sciences, Section of Nephrology, University of Modena and Reggio Emilia, Modena, Italy

The manuscript is improved after the revision. You have provided an updated version of the article that fulfills my requests.

 In conclusion, despite some important technical limitations in the diagnosis of Mucormycosis, the text can be useful in the differential diagnosis of gastric ulcers in an area of Helicobacter pylori endemicity like Nepal.

I have read this submission. I believe that I have an appropriate level of expertise to confirm that it is of an acceptable scientific standard.

# Peer review: Reviewer response for version 1

**DOI:** 10.21956/wellcomeopenres.16394.r34802

**Published:** 2019-03-20

**Authors:** M. Veronica Dioverti

**Affiliations:** 1 John Hopkins University, Baltimore, MD, USA

The authors presented a case of a 57 yo immunocompetent patient that presented with upper GI bleed, diagnosed with H pylori and latter with mucormycosis based on path. She was treated with antifungals alone. There is no mention of evaluation for surgical intervention, which is mainstay therapy for this invasive fungal infection.

 Overall, grammar needs to be further reviewed and corrected (probably needs re-writting); for example: "A 57 years old, smoker female from Sindhuli, a hilly district of Nepal, with no known comorbidities, presented to the Emergency Department of Patan Hospital, Lagankhel, Lalitpur in April of 2018 with one day history of two episodes of bright red blood-mixed vomitus (around 500 ml per episode), but she had dyspepsia, malena and fatigability for 4 months duration. On assessment, she was pale with blood pressure of 90/50 mmHg and heart rate of 130 beats per minute" should read: A 57 year old female from Sindhuli, Nepal, no known medical comorbidities other than smoking, presented to the emergency department with two episodes of hematemesis over 24 hours. She had a 4-month history of dyspepsia, melena and fatigability. On physical exam, she was pale, blood pressure was 90/50 mmgHg with a heart rate of 130 beats per minute"

 "Laboratory parameters with normal ranges in parenthesis, are as follow:"

Question #1 - Background history/case progression:

- Histopathology: granulation tissue suggesting fungal invasion? Was there fungal invasion or no? This should be discernable on path, there should be clear necrosis and vascular invasion in order to make a diagnosis of invasive mucor.
- Was surgical intervention entertained? This is a crucial component of mucormycosis management 
- Was posaconazole level checked? This is important for treatment follow up

Question #2:

 Physical exam/labs: only physical exam comments were that patient was "pale" and they provided blood pressure/heart rate. How about abdominal pain, guarding? Any other ominous signs/symptoms? Was imaging obtained? Oftentimes, mucor will invade nearby tissue. For laboratory, was there a culture sent? In terms of treatment follow up, did she tolerate high doses of amphotericin? Was a posaconazole level checked prior to stopping amphotericin?

Question #3:

 Authors should extend discussion to include how were other patients managed? Typically, management includes surgery. Duration of antifungal therapy is typically longer that 1 month, indicating that perhaps this was not the correct diagnosis? How long were the other case reported treated for?

Question #4: 

 More details need to be included as mentioned above

I have read this submission. I believe that I have an appropriate level of expertise to state that I do not consider it to be of an acceptable scientific standard, for reasons outlined above.

## References

[1] DiovertiMVCawcuttKAAbidiMSohailMRWalkerRCOsmonDR: Gastrointestinal mucormycosis in immunocompromised hosts. *Mycoses*.2015;58(12) : 10.1111/myc.12419 714-8 10.1111/myc.12419 26456920

# Response

**Published:** 2019-03-23

**Authors:** Sudeep Adhikari

**Affiliations:** Patan Academy of Health Sciences, Nepal

Thank you for your constructive feedback to our manuscript.

 The grammar has been reviewed and corrected as suggested by the reviewer.

Question #1 - Background history/case progression:

- Histopathology: granulation tissue suggesting fungal invasion? Was there fungal invasion or no? This should be discernable on path, there should be clear necrosis and vascular invasion in order to make a diagnosis of invasive mucor.
- Was surgical intervention entertained? This is a crucial component of mucormycosis management 
- Was posaconazole level checked? This is important for treatment follow up

Response to question 1

- In the histopathology examination there was no evidence of fungal invasion, as necrosis and vascular invasion were not present. Changes have been made in the revised manuscript.
- Surgical intervention was not done, as our patient had early form of the disease. Case reports where medical therapy alone was used for the management, have been cited in the revised manuscript. (1,2)
- The need of overlapping amphotericin and posaconazole before stopping amphotericin till the therapeutic level of posaconazole is achieved has been discussed in the revised manuscript. (3) But the unavailability of the test of blood level of posaconazole in our setup hindered the use of the standard management practice in our case.

Question #2:

 Physical exam/labs: only physical exam comments were that patient was "pale" and they provided blood pressure/heart rate. How about abdominal pain, guarding? Any other ominous signs/symptoms? Was imaging obtained? Oftentimes, mucor will invade nearby tissue. For laboratory, was there a culture sent? In terms of treatment follow up, did she tolerate high doses of amphotericin? Was a posaconazole level checked prior to stopping amphotericin?

 Response to question 2

- Other physical examinations were unremarkable, including the abdominal exam.
- Abdominal imaging to exclude the invasion of nearby tissue was not done, as our patient had non-invasive form of the disease.
- Confirmation of the infection via culture was not done, because this test is not readily available in our setup.
- Our patient had no complication on using amphotericin B. The hospital stay was uneventful.
- Posaconazole level testing is not available in our setup.

Question #3:

 Authors should extend discussion to include how were other patients managed? Typically, management includes surgery. Duration of antifungal therapy is typically longer that 1 month, indicating that perhaps this was not the correct diagnosis? How long were the other case reported treated for?

 Response to question 3

- Case reports of using medical therapy alone without the surgery, have been included in the discussion.
- Our patient received posaconazole for 2 months only due to financial constraints. But she had clinical as well as histopathological remission from the disease. One case was treated for 6 months with antifungal therapy. (1)

Question #4: 

 More details need to be included as mentioned above

 Response to question 4

- Details have been added as suggested.

**References**

1.  Alfano G, Fontana F, Francesca D, Assirati G, Magistri P, Tarantino G, Ballarin R, Rossi G, Franceschini E, Codeluppi M, Guaraldi G, Mussini C, Di Benedetto F, Cappelli G: Gastric Mucormycosis in a Liver and Kidney Transplant Recipient: Case Report and Concise Review of Literature. *Transplant Proc*. 2018; **50** (3): 905-909
2. Tathe SP, Dani AA, Chawhan SM, et al.: Gastric mucormycosis: Diagnosis by imprint cytology. *Diagn Cytopathol.* 2016;44(10):820–822. 27321416 10.1002/dc.23518
3. Dioverti MV, Cawcutt KA, Abidi M, Sohail MR, Walker RC, Osmon DR: Gastrointestinal mucormycosis in immunocompromised hosts. *Mycoses*. 2015; **58** (12): 714-8 PubMed Abstract | Publisher Full Text 

# Peer review: Reviewer response for version 1

**DOI:** 10.21956/wellcomeopenres.16394.r34951

**Published:** 2019-03-13

**Authors:** Gaetano Alfano

**Affiliations:** 1 Surgical, Medical and Dental Department of Morphological Sciences, Section of Nephrology, University of Modena and Reggio Emilia, Modena, Italy

Adhikari et al. describe an interesting case of gastric mucormycosis in an immunocompetent patient coming from an area where H. Pylori infection is endemic (Nepal). The case is singular because histological examination of the biopsy specimen obtained from the gastric ulcer showed a confection of Mucormycetes and H. Pyroli. Additionally, the case underlined the importance of histologic examination in the early diagnosis of Mucormycosis organisms.

 Some further information should be provided in order to improve the quality of the manuscript:

- The text lacks data regarding the clinical course of the patients after admission. In particular, the authors should describe the change in gastrointestinal symptomatology with the start of the specific therapy.
- Please discuss if culture of gastric mucosal specimen or molecular analysis were performed to identify Mucormycetes
- It is well-known that the management of Mucormycetes infection is based on the prompt institution of appropriate antifungal therapy and surgery. Studies report that surgical treatment has been associated with improved survival when combined with medical treatment (Roden et al., 2005 ^1^; Turner, Soudry, Nayak, & Hwang, 2013 ^2^).

Since the patient did not undergo surgical debridement of the gastric ulcer, the authors should discuss why surgical intervention was not performed.

 On this issue, we published a similar case that was successfully treated with a conservative strategy. After the diagnosis of Mucormycosis, surgery was postponed because the institution of antifungal therapy controlled the disease and resolved the gastrointestinal symptoms progressively (Alfano et al., 2018 ^3^). Although this therapeutic strategy cannot be generalized in absence of randomized controlled trial, antifungal therapy may be considered a first attempt in the early stage of a gastric mucormycosis of non-critically ill patients. Authors may underline that further studies are necessary to establish the best treatment of uncomplicated cases of gastric mucormycosis.

I have read this submission. I believe that I have an appropriate level of expertise to confirm that it is of an acceptable scientific standard, however I have significant reservations, as outlined above.

## References

[1] RodenMMZaoutisTEBuchananWLKnudsenTASarkisovaTASchaufeleRLSeinMSeinTChiouCCChuJHKontoyiannisDPWalshTJ: Epidemiology and outcome of zygomycosis: a review of 929 reported cases. *Clin Infect Dis*.2005;41(5) : 10.1086/432579 634-53 10.1086/432579 16080086

[2] TurnerJHSoudryENayakJVHwangPH: Survival outcomes in acute invasive fungal sinusitis: a systematic review and quantitative synthesis of published evidence. *Laryngoscope*.2013;123(5) : 10.1002/lary.23912 1112-8 10.1002/lary.23912 23300010

[3] AlfanoGFontanaFFrancescaDAssiratiGMagistriPTarantinoGBallarinRRossiGFranceschiniECodeluppiMGuaraldiGMussiniCDi BenedettoFCappelliG: Gastric Mucormycosis in a Liver and Kidney Transplant Recipient: Case Report and Concise Review of Literature. *Transplant Proc*.2018;50(3) : 10.1016/j.transproceed.2017.11.036 905-909 10.1016/j.transproceed.2017.11.036 29573830

# Response

**Published:** 2019-03-23

**Authors:** Sudeep Adhikari

**Affiliations:** Patan Academy of Health Sciences, Nepal

Thank you for your constructive feedback.

**Question**- The text lacks data regarding the clinical course of the patients after admission. In particular, the authors should describe the change in gastrointestinal symptomatology with the start of the specific therapy.

**Response**- Our patient's initial symptoms were resolved during the therapy with antifungal agents. This has been added in the mansucript.

**Question- **Please discuss if culture of gastric mucosal specimen or molecular analysis were performed to identify Mucormycetes

**Response**- Culture of gastric mucosal specimen or molecular analysis were not performed as these tests are not readily available in our setup.

**Question- **It is well-known that the management of Mucormycetes infection is based on the prompt institution of appropriate antifungal therapy and surgery. Studies report that surgical treatment has been associated with improved survival when combined with medical treatment (Roden et al., 2005 ^1^; Turner, Soudry, Nayak, & Hwang, 2013 ^2^).Since the patient did not undergo surgical debridement of the gastric ulcer, the authors should discuss why surgical intervention was not performed.

 On this issue, we published a similar case that was successfully treated with a conservative strategy. After the diagnosis of Mucormycosis, surgery was postponed because the institution of antifungal therapy controlled the disease and resolved the gastrointestinal symptoms progressively (Alfano et al., 2018 ^3^). Although this therapeutic strategy cannot be generalized in absence of randomized controlled trial, antifungal therapy may be considered a first attempt in the early stage of a gastric mucormycosis of non-critically ill patients. Authors may underline that further studies are necessary to establish the best treatment of uncomplicated cases of gastric mucormycosis.

**Response- **The surgical intervention is one important component of management. But we did not opt for the surgical management as our patient had early non-invasive form of the disease. Examples of cases where the early disease had been manged by antifungals alone have given in the revised manuscript.
